# Molar pregnancy in cesarean section scar: A case report

**DOI:** 10.4274/tjod.26878

**Published:** 2017-12-30

**Authors:** Elif Gülşah Dağdeviren, Rıza Dur, Erdem Fadıloğlu, Erhan Demirdağ, Çağatayhan Öztürk, Metin Altay

**Affiliations:** 1 University of Health Sciences, Etlik Zübeyde Hanım Women’s Health Training and Research Hospital, Department of Obstetrics and Gynecology, Ankara, Turkey; 2 Kilis State Hospital, Clinic of Women’s Health, Kilis, Turkey; 3 TOBB University of Economics and Technology Faculty of Medicine, Department of Obstetrics and Gynecology, Division of Women’s Health, Ankara, Turkey

**Keywords:** Cesarean scar pregnancy, molar pregnancy, management

## Abstract

Cesarean scar ectopic pregnancies and molar pregnancies are two very rare obstetric pathologies. In both cases, serious morbidities are involved that require careful management. The coexistence of the two clinical conditions is far less common and there are a limited number of cases in the literature. In this case report, a 34-year-old patient with previous cesarean section was diagnosed as having a molar pregnancy in a cesarean scar through ultrasonography. The patient was asymptomatic at that time. Ultrasonography revealed a protruding mass at the cesarean section and her human chorionic gonadotropin level was measured as 59.705 mIU/mL. Due to the risk of severe bleeding, cesarean section scar excision and revision were performed via laparotomy after counselling the patient. Removal of all trophoblastic tissue was observed as a result of the frozen pathology and the operation was terminated. After the definite pathology result came as a complete molar pregnancy, the patient was followed up according to molar pregnancy follow-up protocols and cured completely. Despite the alternative treatment options (methotrexate application, curettage, uterine artery embolization) in such patients, the decision for surgery was made after counselling the patient. In this very rare clinical condition, patients should be closely monitored and the appropriate treatment option should be applied as soon as possible, taking into consideration the bleeding risks of both pathologies.

## INTRODUCTION

Molar pregnancy is mostly seen in the uterine cavity with a frequency of 1/10000. Ectopic pregnancy (EP) is seen more frequently with an overall frequency of 20/1000, and mostly located in the salpinx. Despite the rarity and different clinical spectrum of the diseases, it may be seen together as a very rare entity, which has been reported with a incidence of one per million pregnancies^(1)^.

Here in, we report a case of molar pregnancy in a cesarean section scar that was diagnosed and managed with surgery at our clinic.

## CASE REPORT

**Figure 1:**
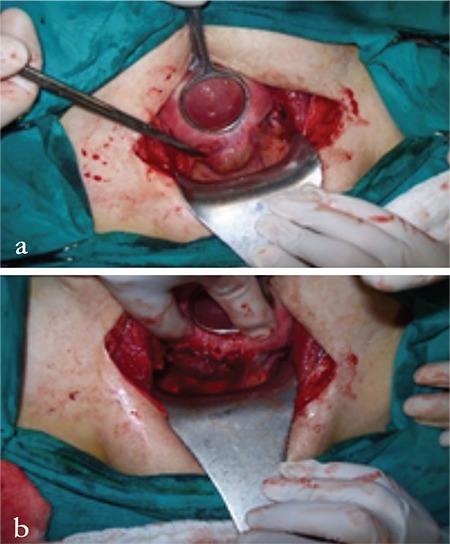
(a) Protruted mass at the cesarean scar (b) Mass reaching the right corner of the cesarean section scar

A woman aged 34 years with a history of one vaginal delivery and one cesarean delivery was hospitalized in our clinic with a diagnosis of cesarean scar EP. The patient had no symptoms at that week of pregnancy. The gestation was calculated as 5 weeks according to the last menstrual period. Transvaginal ultrasound revealed a material in the cesarean scar, which reached the uterine serosa and protruded from anterior uterine wall with dimensions of 28x24 mm. The human chorionic gonadotropin (hCG) level was measured as 59.705 mIU/mL. The patient was suspected as having a molar pregnancy as an initial diagnosis and the possible medical and surgical options were presented to the patient. A surgical approach was chosen as the primary approach and the patient underwent surgery after we acquired informed consent. During the operation, 50x40 mm of pregnancy material was observed in the old cesarean section. After dissecting the bladder from the peritoneum, EP material was seen as a whole reaching the right corner of the old scar (Figure 1).

**Figure 2:**
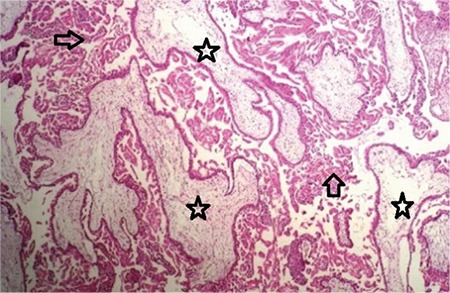
Hydropic villi surrounded completely by proliferative trophoblasts. Arrows indicating proliferating trophoblasts and (*) indicating hydropic villi

The old cesarean scar was incised to reveal the extensions of the molar tissue. Whole trophoblastic tissue was excised through a wedge resection reaching the normal tissue both in the upper and lower segments. The incision was repaired using a double-layer suture after ensuring that no trophoblastic tissue remained. The material was evaluated via frozen section because of the suspicion of molar pregnancy. The result was reported as complete molar pregnancy with negative surgical borders (Figure 2).

The hCG level was 7049 mIU/mL on the second postoperative day. The patient was discharged with hCG follow-up according to molar pregnancy follow-up protocols. The hCG level was 3.2 mIU/mL at the first postoperative month and negative at the first 6-month follow-up. Informed consent was acquired from the patient to publish this case report.

## DISCUSSION

EP is a complication of pregnancy in which the embryo attaches to sites beyond the endometrium, mostly the tuba uterina. Patients mostly admit with vaginal bleeding or severe abdominal pain; EP may also be diagnosed in patients who have no symptoms. Beyond some rare forms of EP, cesarean section scar pregnancy is among the rarest form of EP with an incidence of 1:1800 to 1:2216^(2)^. Cesarean scar molar pregnancy is even more rare due to the rarity of the coincidence of these 2 rare conditions. According to a literature search, 3 cases of cesarean scar molar pregnancy have been reported. Molar pregnancies with ectopic implantation were mostly seen in the fallopian tubes according to case series of molar EPs ^(3)^.

The first case was reported by Wu et al.^(4)^ in 2006. The patient was admitted with vaginal bleeding, which was diagnosed as partial molar pregnancy, and a suction curettage was performed. After on going bleeding after one week, the patient was evaluated again and residual molar tissue was observed in the cesarean scar tissue. Secondary suction curettage was performed with ultrasonographic guidance and treatment was completed.

The second case was reported by Michener and Dickinson^(5)^ in a case series in 2009. One of 13 cases of cesarean scar EP was reported as a molar pregnancy. After administration of methotrexate systematically and into the gestational sac, the patient was followed up. At the 10th month, the patient was admitted with vaginal bleeding requiring hysterectomy. After pathologic confirmation of molar tissue in the hysterectomy specimen, the patient was evaluated as having molar pregnancy on the cesarean section scar.

The third case was presented by Ko et al.^(6)^ in 2012. The patient was admitted with suspicion of retained tissue after pregnancy termination at another clinic for a 7-week pregnancy. Transvaginal ultrasound revealed a suspected molar pregnancy in the cesarean section scar and for histopathologic confirmation, suction curettage was performed. Uterine artery embolization was performed for definitive treatment.

Our patient was admitted to our clinic for routine examination and had no symptoms. Cesarean scar EP was the initial diagnosis. Jurkovic et al.^(7)^ defined the diagnostic criteria for cesarean scar EP as follows: a) Empty uterine cavity and cervix; b) Thinning of the myometrial layer between the bladder and gestational sac; c) Determination of peritrophoblastic perfusion around the gestational sac using Doppler sonography; d) Non-changing position of the gestational sac after gentle pressure from a transvaginal ultrasound probe^(7)^. Our case was consistent with all these findings and cesarean scar EP was the initial diagnosis for this patient. The reason for our suspicion of molar pregnancy in this case was the absence of a gestational sac, existence of extending tissue beyond the uterus, the incompatible value of hCG with the gestational week, which was calculated according to the last menstrual period.

In the treatment of cesarean scar pregnancies, systemic or direct methotrexate admission, wedge resection by laparotomy or laparoscopy, dilatation and curettage, curettage by hysteroscopy, and uterine artery embolization or a combination of these modalities are used^(8)^. Ultrasound-guided suction curettage is accepted as a reliable first-line treatment^(9)^. Suction curettage alone or in combination with other medical interventions has been evaluated as successful according to complications and success rates in case series^(10)^. Cesarean scar pregnancies may also result a high burden of maternal morbidities including severe hemorrhage, early uterine rupture, and hysterectomy with expectant management^(11)^. These pregnancies should be diagnosed carefully to manage patients with minimal morbidities. hCG levels, myometrial thickness, and gestational week must be evaluated to determine the proper approach to minimize morbidities^(12)^.

The decision for laparotomy was made with the suspicion of gestational trophoblastic disease and the high risk of bleeding and perforation. Local methotrexate administration has been reported as a more risky treatment modality for cesarean scar EPs ^(5)^.

Cesarean scar pregnancies are usually identified with ultrasonography and diagnosis maybe delayed. Cesarean section molar pregnancy is a challenging diagnosis and hard to diagnose correctly preoperatively, mostly due to its rarity. Pregnancy localizations should be determined early in pregnancies of patients with past uterine scar or cesarean history and cesarean scar pregnancy should be among our differential diagnoses in such risky pregnancies. The factors mentioned in our case and other cases must be kept in mind so as to acquire the correct diagnosis in these rare cases. Early diagnosis and treatment may be life-saving in such rare cases.

## References

[1] Gillespie AM, Lidbury EA, Tidy JA, Honcock BW (2004). The clinical presentation, treatment and outcome of patient diagnosed with possible ectopic molar gestation. Int J Gynaecol Cancer.

[2] Seow KM, Huang LW, Lin YH, Lin MY, Tsai YL, Hwang JL (2004). Cesarean scar pregnancy: issues in management. Ultrasound Obstet Gynecol.

[3] Yamada Y, Ohira S, Yamazaki T, Shiozawa T (2016). Ectopic Molar Pregnancy: Diagnostic Efficacy of Magnetic Resonance Imaging and Review of the Literature. Case Rep Obstet Gynecol.

[4] Wu CF, Hsu CY, Chen CP (2006). Ectopic molar pregnancy in a cesarean scar. Taiwan J Obstet Gynecol.

[5] Michener C, Dickinson JE (2009). Cesarean scar ectopic pregnancy: a single centre case series. Aust N Z J Obstet Gynaecol.

[6] Ko JK, Wan HL, Ngu SF, Cheung VY, Ng EH (2012). Cesarean scar molar pregnancy. Obstet Gynecol.

[7] Jurkovic D, Hillaby K, Woelfer B, Lawrence A, Salim R, Elson CJ (2003). First-trimester diagnosis and management of pregnancies implanted into the lower uterine segment Cesarean section scar. Ultrasound Obstet Gynecol.

[8] Liu G, Wu J, Cao J, Xue Y, Dai C, Xu J, et al (2017). Comparison of three treatment strategies for cesarean scar pregnancy. Arch Gynecol Obstet.

[9] Özcan HÇ, Uğur MG, Balat Ö, Sucu S, Mustafa A, Bayramoğlu Tepe N, et al (2017). Is ultrasound-guided suction curettage a reliable option for treatment of cesarean scar pregnancy? A cross-sectional retrospective study. J Matern Fetal Neonatal Med.

[10] Özdamar Ö, Doğer E, Arlıer S, Çakıroğlu Y, Ergin RN, Köpük ŞY, et al (2016). Exogenous cesarean scar pregnancies managed by suction curettage alone or in combination with other therapeutic procedures: A series of 33 cases and analysis of complication profile. J Obstet Gynaecol Res.

[11] Calì G, Timor-Trisch IE, Palacios-Jaraquemada J, Monteaugudo A, Buca D, Forlani F, et al (2017). Outcome of Cesarean scar pregnancy: a systematic review and meta-analysis. Ultrasound Obstet Gynecol.

[12] Polat I, Ekiz A, Acar DK, Kaya B, Ozkose B, Ozdemir C, et al (2016). Suction curettage as first line treatment in cases with cesarean scar pregnancy: feasibility and effectiveness in early pregnancy. J Matern Fetal Neonatal Med.

